# Gastric Leiomyoma Near the Gastroesophageal Junction Causing Massive Gastrointestinal Bleeding

**DOI:** 10.7759/cureus.48374

**Published:** 2023-11-06

**Authors:** Ashton White, Jessica Sikora, Abdul Thannoun, Basem Soliman

**Affiliations:** 1 Department of Surgery, Texas Tech University Health Sciences Center, Amarillo, USA; 2 Department of Gastroenterology, Texas Tech University Health Sciences Center, Amarillo, USA

**Keywords:** abdominal tumor, gastric cardia, submucosal tumor, robotic-assisted surgery, gastrointestinal bleeding, gastroesophageal junction, leiomyoma

## Abstract

Leiomyomas are rare, slow-growing submucosal tumors originating from smooth muscle cells. They are typically benign when found in the gastrointestinal tract, and they present no risk for recurrence or metastasis. In this report, we describe the case of a 64-year-old female patient presenting with severe anemia, generalized fatigue, and intermittent dark tarry stools and who was found to have a bleeding gastric cardia mass one centimeter distal to the gastroesophageal junction (GEJ) on abdominal computed tomography and confirmed with an esophagoduodenoscopy. Robotic-assisted laparoscopic wedge resection of the stomach with partial resection of the gastrohepatic ligament was performed to resect the mass. Histopathological examination revealed positivity for smooth muscle actin (SMA) and H caldesmon, consistent with a leiomyoma. In this report, we discuss this patient’s clinical presentation, the method of mass resection, the importance of mass location in choosing a surgical approach for resection, and differential diagnoses for this case.

## Introduction

The gastrointestinal (GI) tract is an uncommon location for mesenchymal, smooth muscle, and neurogenic tumors. GI stromal tumors (GISTs) are the most common to find in the GI tract, followed by smooth muscle and neurogenic tumors [1]. Leiomyomas fall under the subset of smooth muscle neoplasms. They are rare, slow-growing submucosal tumors originating from smooth muscle cells that predominantly occur in the esophagus, colon, and rectum. When found in the GI tract, they are benign, and with resection, there is no risk for recurrence or metastasis [2]. If mitotic activity is present on the histological examination, malignancy should be considered. Below, we describe the case of a 64-year-old female presenting with severe anemia who was found to have a mass in the gastric cardia near the gastroesophageal junction (GEJ) that was diagnosed as a gastric leiomyoma after resection and immunohistological processing.

## Case presentation

**Figure 1:**
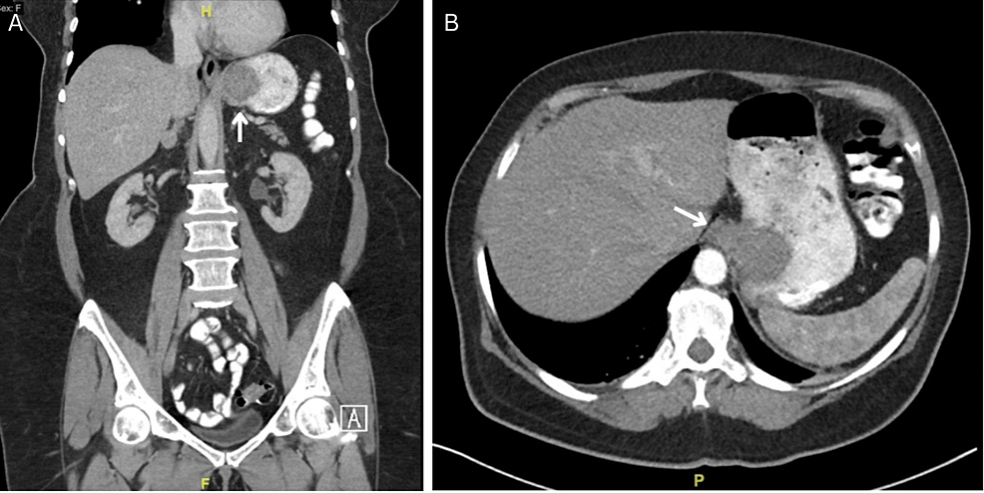
CT Imaging

A 64-year-old female patient presented with a medical history of hypothyroidism treated with levothyroxine and severe anemia treated with iron supplementation. The patient stated that over the past few months, she had been experiencing generalized weakness, fatigue, and dizziness with associated shortness of breath and intermittent episodes of dark and tarry-appearing stools. She denied heartburn, nausea, vomiting, and gastroesophageal reflux disease (GERD). She was afebrile with her main symptom being epigastric pain, with mild epigastric tenderness on examination. She was hemodynamically stable with an oxygen saturation of 96% on room air. Laboratory testing completed on admission showed a hemoglobin concentration of 6.6 gm/dL, and she received two units of RBCs (Table 1). An abdominal computed tomography (CT) scan of the abdomen and pelvis with oral and intravenous contrast revealed an intraluminal gastric cardia mass 1 cm distal to the GEJ measuring 3.9 x 3.9 x 3.8 cm with mild thickening of the posterior gastric fundal wall and gastric lymphadenopathy in the perigastric lymph nodes (Figure 1). An esophagoduodenoscopy (EGD) was performed that confirmed a large polypoid, pedunculated, and bleeding mass. 

**Table 1:** Laboratory Values

Lab Values	Patient Values	Reference Ranges
Hemoglobin (gm/dL)	6.6	12-16
Hematocrit (%)	20	36-46
WBC (x10e3/mcL)	9.8	4.5-11
Platelets (x10e3/mcL)	247	130-400
Sodium (mmol/L)	136	135-145
Potassium (mmol/L)	3.3	3.4-5
Albumin (gm/dL)	1.7	3.1-4.3
Alkaline phosphatase (U/L)	47	30-100

**Figure 2:**
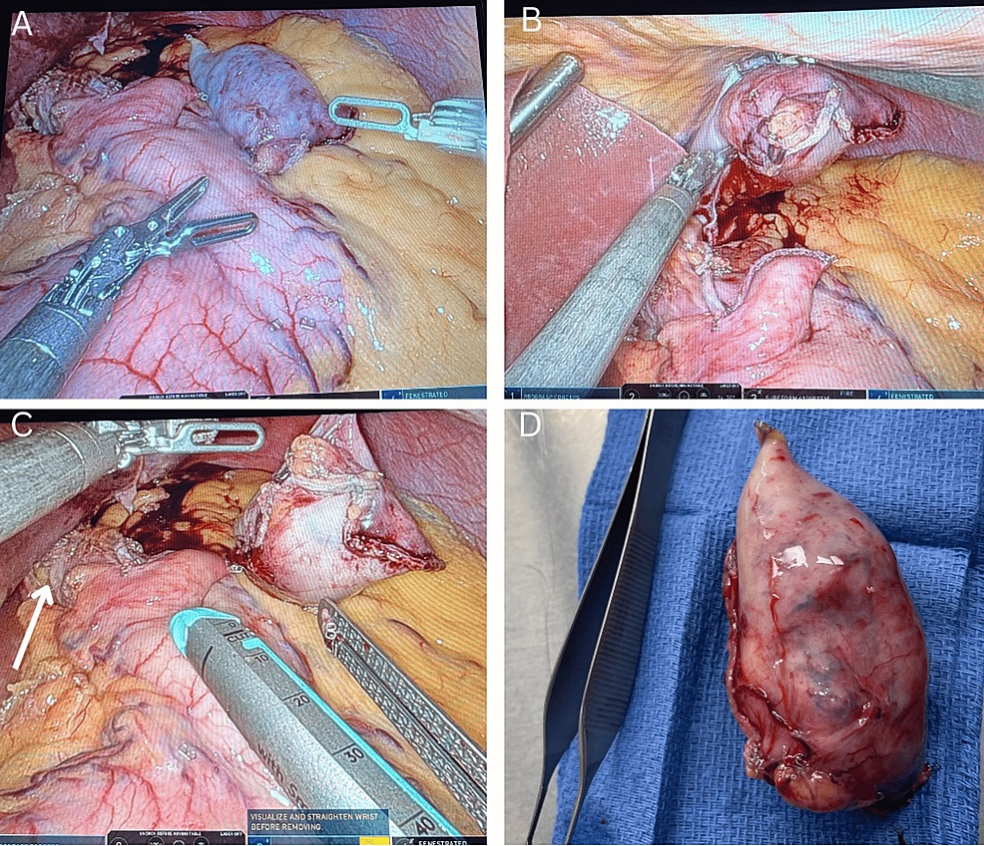
Intraoperative Images of the Gastrectomy

After obtaining informed consent, the patient was taken to the operating room for robotic-assisted laparoscopic partial gastrectomy. Using a 5 mm Optiview 0 camera, the ports were placed in the upper abdomen. After establishing the pneumoperitoneum, a diagnostic laparoscopy was performed that showed no evidence of peritoneal metastasis, liver lesions, or visible ascites or lesions. The robot was docked, and our attention was directed to the left upper quadrant, which showed a visible bulge in the area of the gastric cardia with no associated serosal abnormalities. Using a vessel sealer, the short gastric vessels connected to the fundus of the stomach were taken down. Given the proximity of the mass to the GEJ (labeled with a white arrow in Figure 2C), minimal hiatal dissection was done to gain some laxity and mobilization of the stomach in that area. A 46 French bougie was placed into the stomach at this moment to secure the patency of the gastroesophageal junction. Using a grasper, the mass was identified and held toward the abdominal wall. Using a green load 60 mm SureForm robotic stapler, we stapled across the anterolateral portion of the cardia and two additional green load 60 mm SureForm staples were used to complete the transection of the gastric cardia at the base of the mass (Figure 2). Wedge resection of the stomach including partial resection of the gastrohepatic ligament was performed. Hemostasis was achieved and confirmed. The patient tolerated the procedure well without any complications, and she was transferred to the PACU in stable condition.

Upon completion of the procedure, the specimen was taken to pathology for histological processing. Pathology revealed a 5.5 x 3.4 x 2.9 cm submucosal mass with negative margins, and 0 of 3 lymph nodes were harvested. Sectioning the mass reveals a tan-white, whorled, well-demarcated cut surface. Immunohistochemical stains demonstrate positivity in neoplastic cells for smooth muscle actin (SMA) and H caldesmon with patchy nonspecific staining for DOG1 and CD117. The neoplastic cells are negative for CD34. These findings were consistent with the diagnosis of leiomyoma.

The patient tolerated the procedure well and was started on a clear liquid diet postoperative day one. She advanced to a full liquid diet on postoperative day two and was discharged home with instructions to advance to a soft diet in three days. The patient returned to the clinic for follow-up two weeks and two months postoperatively. She was still tolerating her diet well without nausea or vomiting and denied further bloody or black bowel movements. The plan is to follow up with this patient in one year for history, physical exam, and CT scan. The overall prognosis is good for this patient.

## Discussion

A gastric leiomyoma is a submucosal growth that accounts for 2.5% of gastric tumors. Some cases are clinically evident because of bleeding from ulceration of the overlying gastric mucosa. However, several pathologies present with similar clinical symptoms and tumor locations. It is important to differentiate between these pathologies to identify the correct diagnosis and subsequent treatment.

One important differential to consider is gastric adenocarcinoma, which can arise from heterotrophic gastric glands (HGG) and can present with anemia and evidence of upper gastrointestinal bleeding [3]. It is difficult to differentiate between a malignant adenocarcinoma and a benign leiomyoma based only on a CT scan, and histopathologic testing is diagnostic. GISTs are another differential to consider. GISTs are the most common malignant mesenchymal tumors to find in the gastrointestinal tract with an incidence of 10/million/year. They originate from the muscularis propria of the gastrointestinal wall and stain positive for marker CD34 [4]. This is in contrast to leiomyoma, which has a benign classification, smooth muscle submucosal tissue origin, and a negative staining pattern for CD34. Another differential pathology is a gastric schwannoma. Schwannomas are predominantly found in the subcutaneous tissues of the extremities. However, if found in the gastrointestinal tract, the stomach is the prevalent site. Similar in nature to the smooth muscle leiomyoma of our patient, gastric schwannomas primarily occur in the gastric submucosa. Fourteen percent of gastric schwannomas present with gastrointestinal bleeding. Homogeneous attenuation is characteristic of schwannomas, which contrasts with the low attenuation found in leiomyomas. A strong expression of S100 with an absence of DOG1 would have raised suspicion for a diagnosis of gastric schwannoma [5]. Finally, gastric lipomas comprise 1-3% of benign stomach tumors and would be considered a rare differential in a patient with a history of gastrointestinal bleeding [6,7]. 

The location of our patient’s leiomyoma at the GEJ has particular importance when considering postsurgical complications and the efficacy of surgical approaches. Significant bleeding has been reported after endoscopic tumor removal near the GEJ because of the well-developed submucosal vascular network in that area [8]. Resection of leiomyoma of the GEJ has been traditionally performed via laparotomy. However, the surgical view of the lower mediastinum is poor in open surgery as compared to minimally invasive techniques such as laparoscopy and robotics and increases the risk for surgical complications [9]. It is particularly challenging to treat submucosal lesions located at the posterior aspect of the GEJ with a transgastric laparoscopy combined with endoscopic assistance because of a high risk of causing deformity, stricture, or leakage [10]. In 2011, a case study of four patients with giant leiomyomas originating from the posterior aspect of the GEJ was treated with full-thickness endoscopic retroflex dissection. The first patient experienced some degree of air filtration causing symptomatic pneumoperitoneum, and the remaining three patients had prophylactic paracentesis at the end of the endoscopy, but mild subcutaneous emphysema was still observed in these patients. The mean operative time for the endoscopic removals was 180 minutes (range 150-220 minutes). The use of a robotic approach resulted in shorter surgery times and fewer operative complications [11]. The robotic approach should be a key consideration for surgery, as it has many advantages such as improved dexterity and range of motion with the use of multiple arms, better ergonomics, and enhanced visualization of the surgical field and isolation of structures as compared to laparoscopic or open techniques [12].

## Conclusions

Leiomyoma is an important differential to consider in a patient with a history of gastrointestinal bleeding. The resection of a leiomyoma located near the GEJ is challenging, but using a minimally invasive robotic approach should be considered a safe option that will not affect the functional outcome.
